# The international Human Genome Project (HGP) and China’s contribution

**DOI:** 10.1007/s13238-017-0474-7

**Published:** 2017-10-06

**Authors:** Xiaoling Wang, Zhi Xia, Chao Chen, Huanming Yang

**Affiliations:** BGI-China, Shenzhen, 518083 China

*I can*’*t imagine something like this*: *One day* (*the future which is now*) *a pupil asked such a question in class when I taught about the HGP*, “*My teacher, why didn*’*t China do something for the HGP*?” *I would deeply regret for the rest of all my life if my response could only be the following*: “*My kids*, *how can you know*, *ah*, *that time*, *how poor our country was*, *how illiterate our people were*, *how short*-*sighted our decision-makers were*, *and how disappointing our scientists were*, … *we just saw our great nation thus losing another historic opportunity without doing anything*, ……” *No*, *it will never come true*.

— A Chinese middle school teacher (1999)

The HGP is the first and greatest endeavor so far to understand ourselves and all other types of life through the vast international collaboration. It is widely acknowledged as one of the three most important projects in natural sciences of the 20th Century (Lambright, 2002). It is also generally accepted by the scientific community as “the 2nd revolution” in life sciences, following “the 1st revolution” discovering DNA double helix (Sharp, 2014).

## The initiation and globalization of the international HGP

The worldwide discussion about the significance and feasibility, as well as the relevant ethical and social issues, of the HGP began at the beginning of the 1980s. The first official meeting about the HGP was held in 1984 in Utah, USA. The USA became the first country to initiate the HGP in October, 1990.

**Figure 1:**
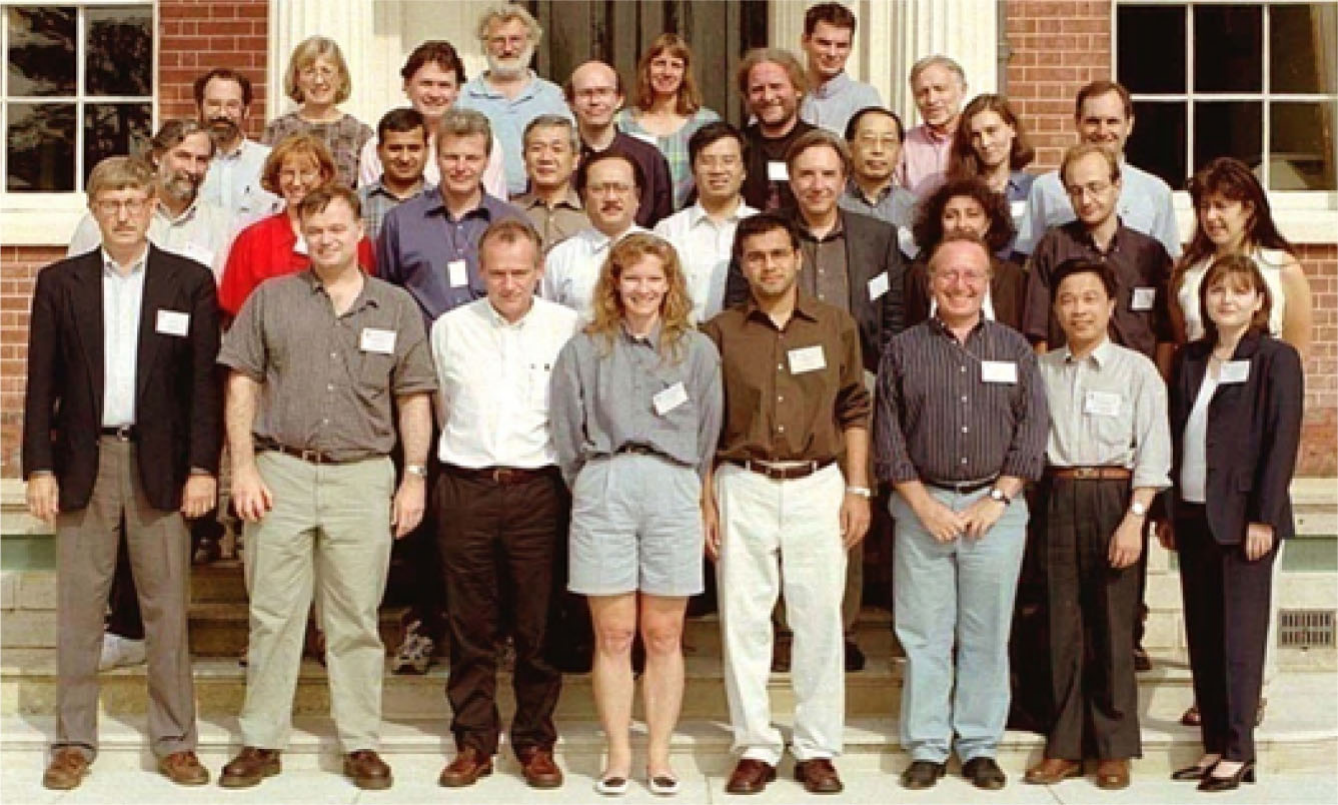
The International Human Genome Sequencing Consortium at the 5th Strategic Meeting, Aug. 31, 1999, Hinxton, UK

The HGP was joined by the UK, France, Germany, and Japan in 1996 at the 1st “International Strategic Meeting on Human Genome Sequencing” (1st ISMHGS, also called “Bermuda Meeting”), in Bermuda, proposed and organized by Dr. Michael Morgan, Director of Welcome Trust, UK. The HGP was further globalized by China’s participation at the 5th Strategic Meeting in Hinxton (Fig. 1), UK, after a cautious discussion and serious defense, and became the “latest contributor” of the HGP and the only developing country in the International Human Genome Sequencing Consortium (https://www.genome.gov/10002109/).

## China’s contribution to the HGP

China was committed to sequencing, assembly, and analysis of a region of approximately 30 cM (centimorgan, a unit of “genetic unit” measuring the size of a genome) on the tip of the short arm of chromosome 3, which was then estimated to account for about 1% of the entire human genome, thus called the “1% Project”, or the “Chinese Chapter of the Human Genome Sequence”, or the “Beijing Region” of the human genome because all of the sequenced overlapping BAC (Bacterial Artificial Chromosome) clones are labeled “Beijing”.

The debates on the HGP and China’s participation were no less than those in the US and other countries since the very beginning. We can now still read from the publications at that time, such as “(the HGP) has been ‘cut as a watermelon’ by big labs and monopolized by a few big companies, we should not be involved in the competition”, as well as “the human genome sequencing is a pseudoscience, … those people arrange the apples on ground and try to convince us it is the sequence of apples on the tree” and so on.

The commitment to the HGP means that China had to complete 500,000 successful Sanger sequencing reactions within six months between October, 1999 and March, 2000. The “1% Project” was officially sponsored by the 863 Project of the Chinese Ministry of Science and Technology (MOST), and followed by China Natural Science Foundation (NSFC) and Chinese Academy of Sciences (CAS). The Chinese Human Genome Consortium (CHGC) was composed of 15 teams from the Northern Center of National Human Genome Center (Beijing), the Southern Center of National Human Genome Center (Shanghai), the Human Genomics Center of the Institute of Genetics, Chinese Academy of Sciences, and BGI, as well as Xi’an Jiaotong University, Southeast China University, and other institutions. BGI was responsible for providing all BAC clones after careful selection of the “seed clones” by means of *in situ* hybridization and other mapping methods, remapping and confirmation of all “extended clones” for all other participating teams, as well as the assembly and filling in both “inter-clone gaps” and “inner-clone gaps” through bioinformatic tools and other techniques.

**Figure 2:**
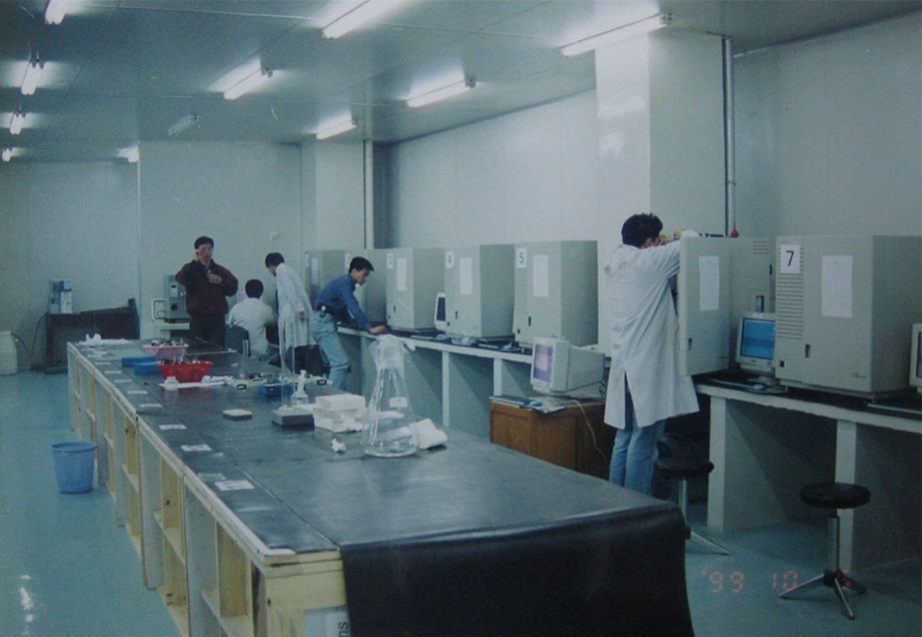
**A lab for the “1% Project”**

**Figure 3:**
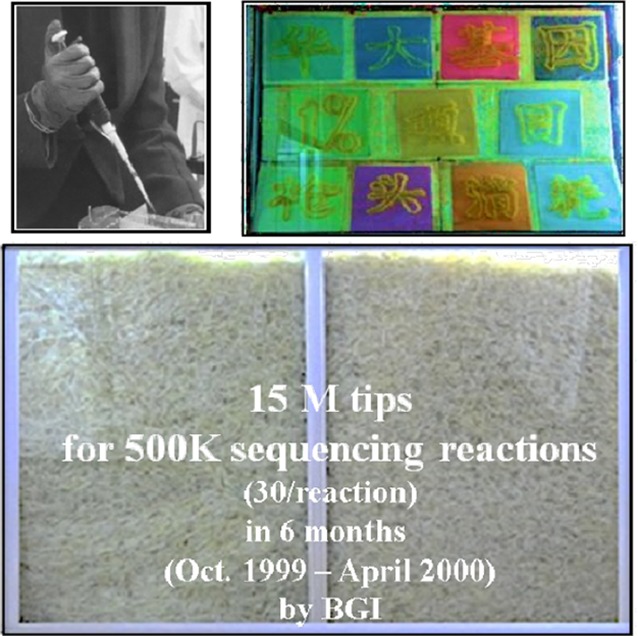
**A small sampling of used tips for the “1% Project”**

Technically, the “1% Project” began almost from “Ground Zero”. Bearing the “for glary of the motherland” and “proving ourselves that we can do others are able to do” in minds, all the participants have a good mastery of all required skills after intensive and strict training in a short time. They developed a pipeline “with Chinese characteristics” both efficient and economic. They even made the working benches with packing boxes, to overcome the difficulties of insufficient funding without any intention to show off (Fig. 2). We can also see many pictures showing how they were working day and night those days. Just take it as an example, more than 15 million of pipetting tips of various size have been used up for the project by means of the technologies that time (Fig. 3).

**Figure 4:**
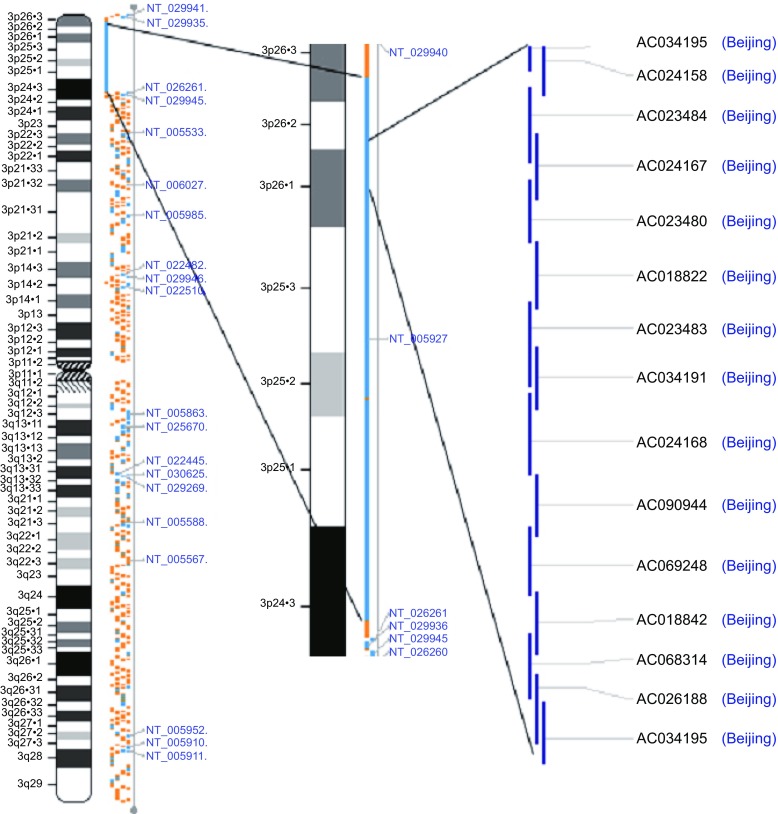
**The “Beijing Region” of the fine map of the human genome sequence**

Finally, through the efforts of all teams, the CHGC submitted 64 Mb (a million of base pairs) raw data for the human genome “draft sequence” before the joint celebration of the “Human Genome Draft Sequence” on 26 June, 1999, and 38 Mb data of “finished sequence” without even a single “gap” in the whole region (Fig. 4) for the paper of the human genome fine sequence published in *Nature* in Oct, 2004 (International Human Genome Sequencing Consortium, 2004). The fine sequence of the human chromosome 3 was finally published in *Nature* in April, 2006 (Muzny et al., 2006).

The international HGP was officially closed with the “Joint Proclamation by the Heads of Government of Six Countries Regarding the Completion of the Human Genome Sequence” on 14 April, 2003 (Box 1) (Administration of George W. Bush, 2003). The former US president and UK prime minister, as well as other state heads, have fully acknowledged the contribution by the Chinese scientists on different occasions.

China’s participation not only improves the international representation of the HGP and made it the first vast internationally collaborative project joined by both developed and developing countries in history, but also marks an important starting point for Chinese scientists to play their important roles on the international science stage as they are doing now.

In addition to the substantial scientific contribution, Chinese scientists made irreplaceable contribution to the international supports by making the UNESCO statement on 7 May, 2000 published supporting the free-sharing principle of human genome sequences, as well as many other efforts. Sir John Sulston, a Nobel Prize Laureate and the British HGP leader, said, “I especially salute the Chinese colleagues, who have contributed so much to the international genome effort … and affirmed its common ownership by all humankind.”

The contribution by China to the HGP is carved on the bronze road of the Chinese Millennium Monument, as one of the important events in Chinese history.

**Box 1:** Joint Proclamation by the Heads of Government of Six Countries Regarding the Completion of the Human Genome Sequence.

## The impacts of the HGP and China’s participation

The HGP came to an end about one decade ago, its impacts on life sciences and bioeconomy can be seen everywhere, and will surely be felt with time going on.

The HGP has cultivated a new field, GENOMICS, which has made almost all branches of the life sciences “omicized”, e.g., proteomics, epigenomics, canceromics, phenomics, and numerous others. Genomics, with “life is in sequences” and “life is digital” as its belief and pillars, is extended to make all phenotypes digitalized, laying foundation for bioinformatics in a new phase of life sciences.

The HGP has fueled the development of a technology, SEQUENCING, which has made life “sequencized” and “digitalized”, thus revolutionized biology and medicine for the 21st Century.

More importantly, the HGP has nurtured a CULTURE of collaboration under the HGP Spirit: “*Owned by All*, *Done by All*, *and Shared by All*”, as proposed by the Chinese scientists and endorsed by the HGP community. The HGP has established a brilliant example of global collaboration for the sake of humanity and scientific advancement, and followed by the International HapMap Project, the International 1000 Genome Project, the International Cancer Genome Project, the Global BioGenome Project, and dozen of other internationally collaborative projects going on now.

The HGP, together with other newly emerging technologies, such as iPS/stem cell and animal cloning, genome editing and writing (“synthetic genomics”), big data, deep-learning and AI (Artifical Intelligence), as well as other-omics, has “*changed biology and biotech forever*” and has brought us a new opportunity for building a more beautiful life world and our confidence of a better future of mankind.
